# How to Improve Speaking Skills in Plastic Surgery Training? Survey in Residents Participants in Pecha Kucha Contest

**Published:** 2018-05

**Authors:** Guillermo Ramos-Gallardo, Gabriel Ángel Mecott-Rivera, R Limon-Cervantes, M García-Pérez, E Rodríguez-Olivares

**Affiliations:** Mexican Society of Plastic, Aesthetic and Reconstructive Surgery, Asociación Mexicana de Cirugía, Plástica, Estética y Reconstrutiva (AMCPER), Mexico

**Keywords:** Speaking skill, Plastic surgery, Training, Pecha Kucha contest

## Abstract

**BACKGROUND:**

Little is known about educational games in Plastic Surgery training. Pecha kucha game has proved to be helpful tool to improve communicative skills. This study survey in resident participants in Pecha Kucha contest assessed how to improve speaking skills in plastic surgery training.

**METHODS:**

In the second edition of Pecha Kucha contest of the Mexican Society of Plastic Surgery, a survey was conducted with the residents to know the utility of this educational game.

**RESULTS:**

Twenty-six residents participated in the survey. Most of them from the Universidad Nacional Autonoma de México. Most of the residents considered it to be a good tool in order to improve communication skills and helpful for their future practice. The amount of time to present an idea was considered enough to express an idea. The most common proportion between words and images was 20-80% in the presentation.

**CONCLUSION:**

Pecha Kucha helped to improve communication skills during residents’ training. We encourage other plastic surgery societies to incorporate educational games in their national and international meetings.

## INTRODUCTION

Aa doctor especially in the field of plastic surgery is exposed to different scenarios where he/she expresses ideas, surgeries, or research work, so in this way he/she needs to discuss and expose his/her thoughts in the most assertive and express way. Little is known about the best way to present articles, classes or even conferences especially in medical school or during residence training. Brief information is given during medical career or residence training, for example in the case of power point presentation, suggestion as seven lines per slide or every line should have seven words is common.^1^

It is not strange that tables and figures with important information are introduced and emphasizes is not strong enough as consequences audience loose interest even with an excellent presentation. Probably plastic surgeons are more visuals and new strategies are mostly focused to retain attention and interest in big auditorium. The important question is if we can do something in residence training to improve speaking skills and how we can introduce better experience in residence program?^2^

Little is known in this matter and future generations of plastic surgeons are growing. In order to improve this situation, we are developing strategies to improve speaking skills. Two different educational games are introduced in the Mexican Society of Plastic Aesthetic and Reconstructive Surgery. One of them is Pecha Kucha, that comes Japanese chit chat or the sound of conversation is a power point presentation style created in 2003 by architects Mark Dytham and Astrid Klein as a way to ensure brevity in the presentation of young designers.^1^ It follows certain rules as 20 slides in total or every slide in should last 20 seconds, in total 6 minutes with 40 seconds. Pecha Kucha has extended in different fields, now in plastic surgery training. The following paper presents the results of the survey done in the second edition of Pechakucha contest in plastic surgery residents’ members of the Mexican Society of Plastic Surgery. 

## MATERIALS AND METHODS

A survey was conducted to plastic surgery residents in the second edition of Pecha Kucha contest in the city of Monterrey, August 20^th^, 2016. The survey consist of 17 questions design to explore if the resident was exposed to a similar experience in medical school or residence training, if they were satisfied with time and rules of the game and to know the perception about how to present papers in plastic surgery meeting. No money was given to answer and we keep confidential the identity of the residents. The survey is shown in the Table 1. 

**Table 1:** Pecha Kucha Survey.

Plastic Surgery Residents Mexican Society of Plastic, Aesthetic and Reconstructive Surgery	
1.	During your medical career did you have any experience in educational games?
2.	Do you think Pechakucha contest will help you in future?
3.	Do you think Pechakucha contest will stimulated learning experience in your training?
4.	Do you think Pechakucha contest will improve your skills to present papers in congress or classes?
5.	How long do you think an idea can be expressed in Plastic Surgery?
6.	What is the relationship that must be between words and images in a slide?
7.	Do you think the contest will give more time for presentation?
8.	Do you think the contest will give less time for presentation?
9.	How long should last average conference in Plastic Surgery meeting?
10.	How long should last main conference in Plastic Surgery meeting?
11.	How long should last research presentation in Plastic Surgery meeting?
12.	During medical school did you receive information about how to present classes?
13.	During medical residence did you receive information about how to present classes?
14.	The topic that you chose was related with aesthetic, reconstructive or different topic?
15.	How many classes do you present at month?
16.	How long classes last in your program?
17.	Why did you choose the topic of your presentation?

## RESULTS

Twenty-six residents participated including 16 males (69.2%) and 8 females (30.7%). The median age was 30.6 years with a range of 28 to 36 years. Most of the participants were in the third year of their training (10 residents: 38%). They came from different medical school in Mexico, private and public. Two of them came from medical school outside Mexico (Bolivia and Dominican Republic). About the universities and plastic surgery trainings in Mexico, only four universities have recognized programs in plastic surgery: Universidad Nacional Autónoma de México (UNAM), Universidad de Guadalajara (UdG), Universidad del Autónoma Estado de México (UAEM) and Universidad Autónoma de Nuevo León (UANL). We received participant from the four universities, being the UNAM the one that sent the most, 12 participants (46%).

About similar experience during medical school, only one resident answered positive. Two residents answered that the contest won´t be positive for their training, 89.5% answered that this type of contest will have positive impact in their training. When we ask about the reason of the answer, the responses were related with improving personal communication, relationship with other residents from other programs and better way to improve analysis and synthesis of information. About the rated of the contest related with their positive impact in their plastic surgery training in scale of 1 to 10, being 1 no helpful and 10 very helpful, the median answer rate was 8.4 (range: 2 to 10). 

When we asked if this experience will improve the ability to present papers, most of them agreed and the rates were in scale of 1 to 10 being 1 not helpful and 10 very helpful, the median was 9. About the balance between images and words in every slide, most of the participants (46%) answered that the best balance would be 20% words and 80% images. All participants agreed that the time to express an idea was good: 6 minutes and 40 second. Related with the right time to present conferences, the participant answered in case of an average presentation in plastic surgery congress the most common answer was from 10 to 20 minutes, main conference more than 20 minutes and to present research work less than 10 minutes. 

Only 15% percent of the participants answered that they received information in medical school about how to present a class, and only two programs explained to resident how to present class. All residents must present classes during their training at least once every month, lasting from 20 minutes to one hour. About their presentation 62% preferred reconstructive topics and most of them chose the topic because they considered it was interesting. 

## DISCUSSION

During medical career and plastic surgery training, most of residents were exposed in different scenarios were talking in public is mandatory. Nowadays, we need to discuss with patients, different treatment options and every time the academic setting is being more competitive. Mastering talking in congress or conference is critical. There are some examples of excellent surgeons that cannot talk in public, where this type of contest can help to improve communicative skills. The question will be if we knew what is the best way to present conferences.^1^

About computer programs, power point is the most common used. It has clear advantages as easy to use and no difference exit between computers. There are some other programs as Prezi that can make presentation more attractive. We observed that it was better experience to use Prezi in steat power point.^2^^,^^3^ Most of us are more visual and attention can be kept in better way. In plastic surgery, it is important to know about surgical technique or pictures before and after, little is explore about statistical analyses or *p* values.^2^^,^^3^

As we are more visual in the case of a big meeting, we can be lost with so many presentations that our attention can decrease time after time. Ideas can be better expressed with images instead of words. If we try to clear difference in results, probably a table can be a good way but if instead of table we present image full of balloons of same color and suddenly one balloon with different color appear that can be best way to keep attention talking about the differences in studies. It is probed that the most attention is keep during the first minutes.^4^

If we lose time at the begging with a big introduction, at the time to present results or main message public can be distracted and retention will decrease. Every person is different, but training with this method can make better speakers, quality that is not exploded in all programs. Pecha kucha has proved to be powerful tool to have better communication skills in other fields.^5^ The important component is the emphasis presented. If incredible slides are not mix with good speaking skills that includes body language, the conference would not have same impact. Pauses and increase of tone should be important component to make emphases in the presentation.^6^

The judges of the contest were not only plastic surgeons. Professionals of communications were invited our main focus is the ability to express ideas following the rule of 20 slides, and every slide should be presented in 20 seconds. Some of the answers of the opened questions mentioned that it was not easy to take idea in 20 seconds and excellent knowledge of the topic must be master. Another important part that was evaluated is the emotional stress that full auditorium can make. It is not easy to handle panic and fear. Without a doubt practice can make perfection.^7^

The main purpose is to master knowledge and to be prepared to express an idea in the best possible way. This type of contest is not main focus in science or research. As Society we count with National Resident Contest, where research projects are presented and the impact of the results is evaluated as well as other scientific points. Discussion of methodology or possible bias of the research are openly exposed. Difference is that we want to promote friendship and relations between residents and programs. At the end, residents were invited to take dinner and play karaoke. We acknowledge efforts that every program is making and we provide to all participants’ scholarship to yearly national meeting of our society.^8^

Unfortunately, during medical school or resident training educational games are not promoted. At the end of the year, Jeopardy is being organized. Besides the good and enjoyable time educational games can improve memory and knowledge. Little is known about the efficacy of this tool. For this reason this type activities can give more information about the best way to improve learning and training experience. Other important factor that is not analyses is the available information that residents can have online.^8^

Youtube, Face book or Twitter can make learning process more easy and attractive. Nowadays, students have more access to information, making professor to be better prepared. Probably Youtube cannot be the best educational system to learn important parts of different surgical procedures. Important efforts from other fields of knowledge, specialties and plastic surgery societies are done in order to have new platforms of learning where educational videos can improve experience and make longer impact in knowledge.^8^

Longer follow up of the participants will help to know if this type of contest helped them to get better scores in Mexican Plastic Surgery Boards and if they get better involved to present papers in congress and to have better practice, academic or private. Pecha Kucha contest was a good experience for majority of participant. They found a helpful contest in order to be better prepared as plastic surgeons and future speakers. The ideal time between images and words is 80% to 20%. This is the first experience to an educational game for most of the participants. Besides, most of programs request classes to their residents, few of them explain how they want the presentation. 

Pecha Kucha contest helped residents to be better prepared and to make better presentations with more information and more visual contest. Residents showed more interest for reconstructive topics. Pecha Kucha contest should be part of educational activities for residence programs making better speakers and exposing speaking to an auditorium. We encourage other plastic surgery societies to get more involved and develop more educational games as Pecha Kucha or Jeopardy in their national and international meetings. 

## CONFLICT OF INTEREST

The authors declare no conflict of interest.

## References

[1] Klentzin JC, Paladino EB, Johnston B, Devine C (2010). Pecha kucha: using “lightning talk” in university instruction. Ref Serv Rev.

[2] Beyer A, Gaze C, Lazicki J (2012). Comparing students’ evaluations and recall for student pechu kucha and power point. JoTLT.

[3] Nguyen H (2015). Student perceptions of the use of pecha Kucha presentations for EFL reading classes. Language Education.

[4] May Tomsett P, Shaw MR (2014). Creative classroom experience using pecha kucha to encourage ESL use in undergraduate business courses: A pilot study. Int Multiling J Contemp Res.

[5] (2011). Communicating competence through pechakucha presentations. J Business Communic.

[6] Valkenburg PM, Peter J, Walther JB (2016). Media effects: Theory and research. Annu Rev Psychol.

[7] Bik HM, Dove ADM, Goldstein MC, Helm RR, MacPherson R (2015). Ten simple rules for ffective online outreach. PLoS Comput Biol.

[8] Bachiller P, Gómez A, Núñez Trujillo P, Ortiz-Caraballo C, Sosa Sánchez E (2014). Pecha kucha politec: trabajos de clase en 6:40 para todos. Acta Symp.

